# Technique of neuromonitoring during pelvic peritonectomy

**DOI:** 10.1515/pp-2020-0132

**Published:** 2020-08-25

**Authors:** Antonio Macrì, Giorgio Badessi, Carmelo Mazzeo, Marica Galati, Eugenio Cucinotta, Vincenzo Rizzo

**Affiliations:** Peritoneal Surface Malignancy and Soft Tissue Sarcoma Program, Messina University Medical School Hospital, Messina, Italy; Department of Human Pathology in Adulthood and Childhood G. Barresi, University of Messina, Messina, Italy; General and Emergency Surgery Unit, Messina University Medical School Hospital, Messina, Italy; Department of Clinical and Experimental Medicine, University of Messina, Messina, Italy

**Keywords:** cytoreductive surgery, HIPEC, intraoperative neuromonitoring, peritoneal surface malignancies, peritonectomy

## Abstract

**Objectives:**

Pelvic peritonectomy can induce anorectal and urogenital dysfunctions. To reduce this type of complications during the procedure, we propose to use intraoperative neuromonitoring (IONM).

**Content:**

Stimulation with a bipolar probe allows the identification of the obturator and ilioinguinal and pudendal nerves. At the end of the cytoreductive surgery, the motor and somatosensory evoked potentials must be evaluated to confirm the preservation of pelvic innervation.

**Summary:**

The use of IONM during pelvic peritonectomy is technically feasible, and it can help to preserve pelvic nerves.

**Outlook:**

Obviously, its definitive value remains to be elucidated.

## Introduction

Cytoreductive surgery (CRS) plus hyperthermic intraoperative chemotherapy (HIPEC) have considerably improved the prognosis of peritoneal surface malignancies (PSMs) [1]. However, after pelvic peritonectomy and en bloc pelvic resection, fundamental phases of this multimodal approach, some patients are known to experience, probably correlated to complexity of peritoneal innervation [2], anorectal and urogenital dysfunctions, which can exert significant impact on the long-term quality of life (QOL) [3]. To reduce this type of complication, we propose to use intraoperative neuromonitoring (IONM), borrowing it from Kneist’s experience [4], [5], [6], that was first introduced this technique in rectal surgery.

## Materials and methods

CRS plus HIPEC are performed as previously described [7]. Anesthesia is induced with 0.5 μg/kg sufentanil (0.5 μg/kg), propofol (2 mg/kg), and atracurium (0.5 mg/kg). Maintenance is achieved with sevoflurane (0.8–1 MAC) in air/O_2_ (FiO_2_ > 0.4, flow 0.5 L/min) and bolus injections of sufentanil (5–10 μg). For muscle relaxation, atracurium is used.

**Figure 1:**
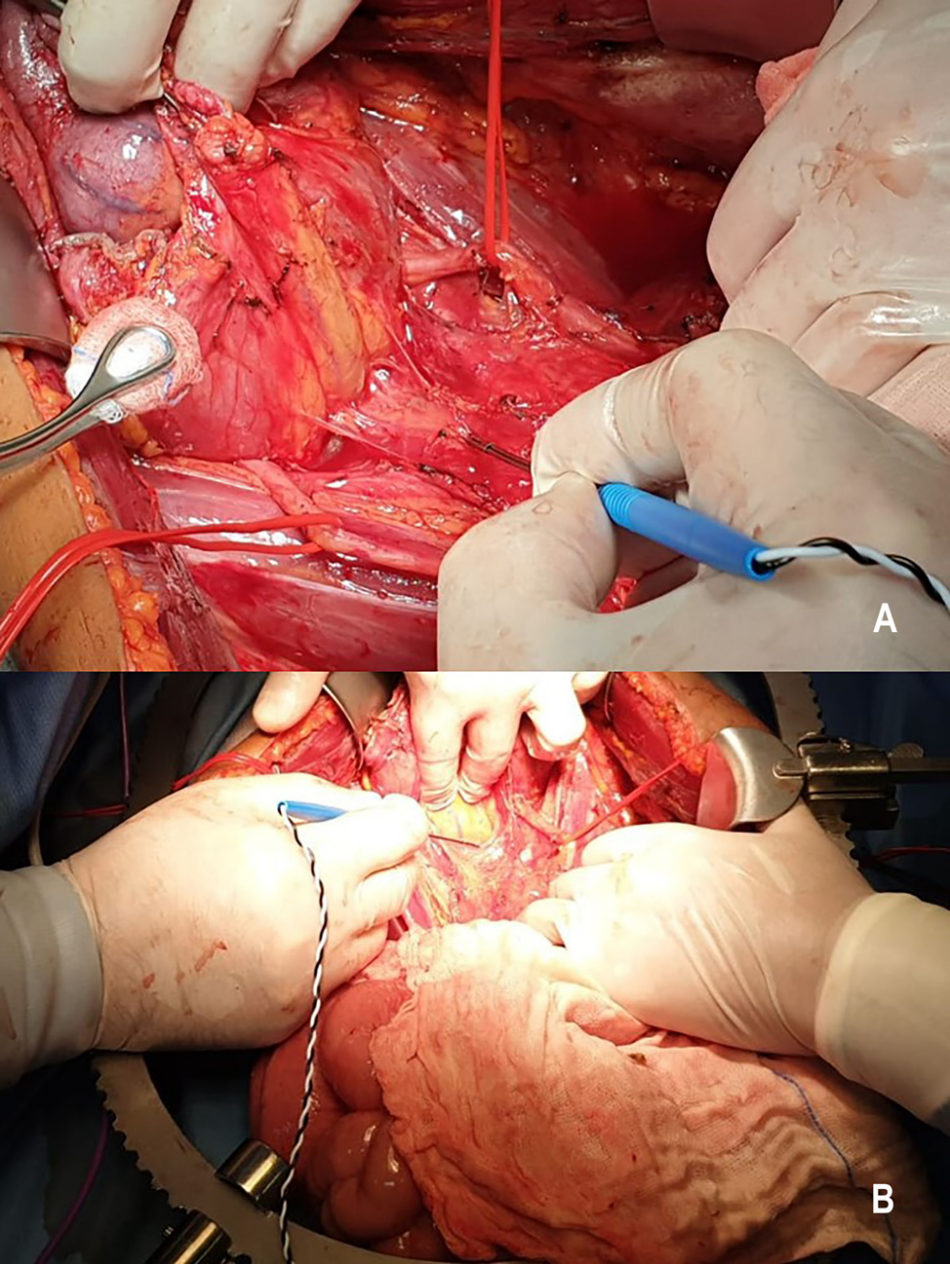
Neurostimulation with a bipolar probe at the level of the sacral promontory (A) and during anterolateral rectal dissection (B).

**Figure 2:**
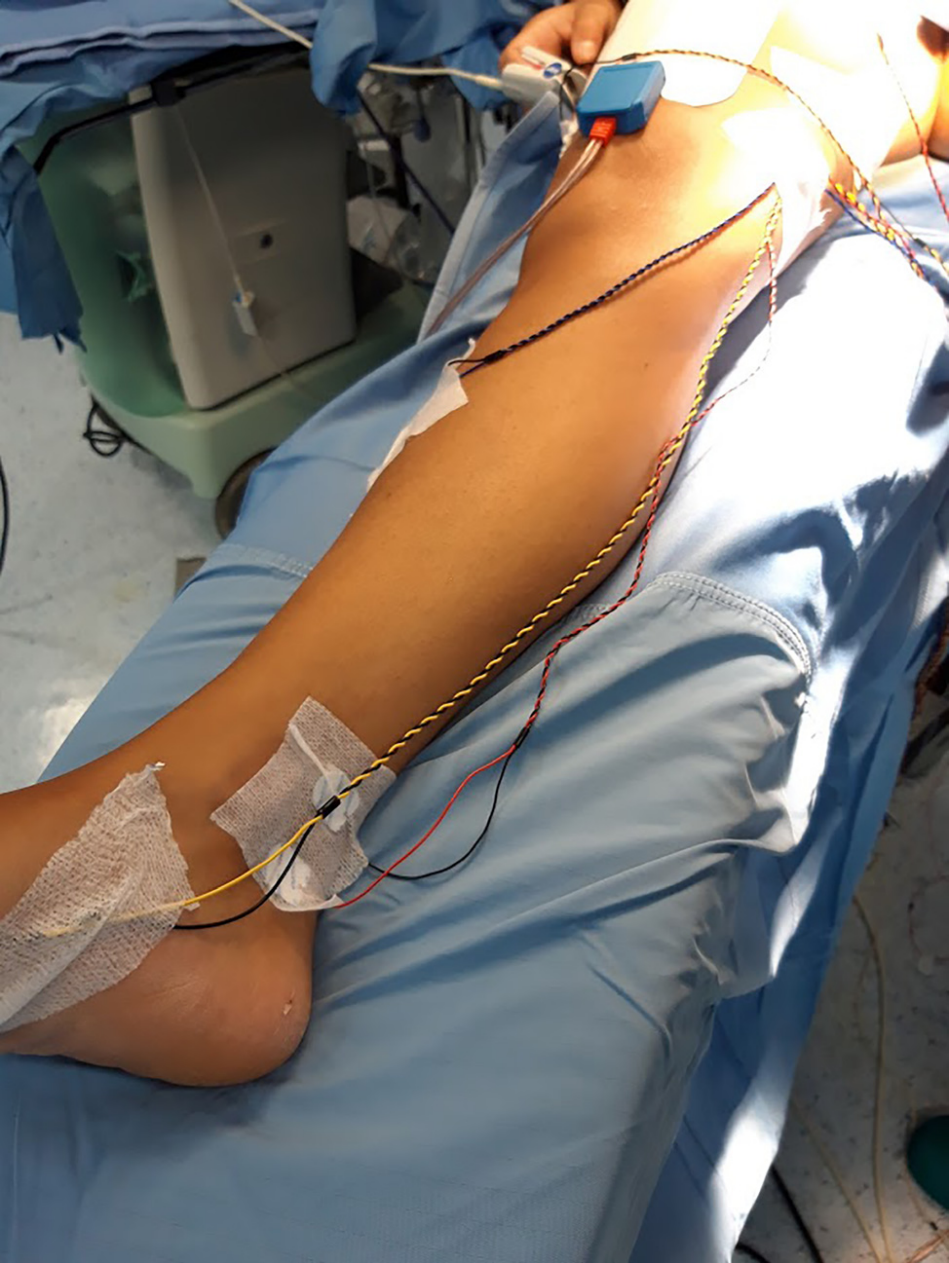
Electrical stimulation of the tibialis nerve.

**Figure 3:**
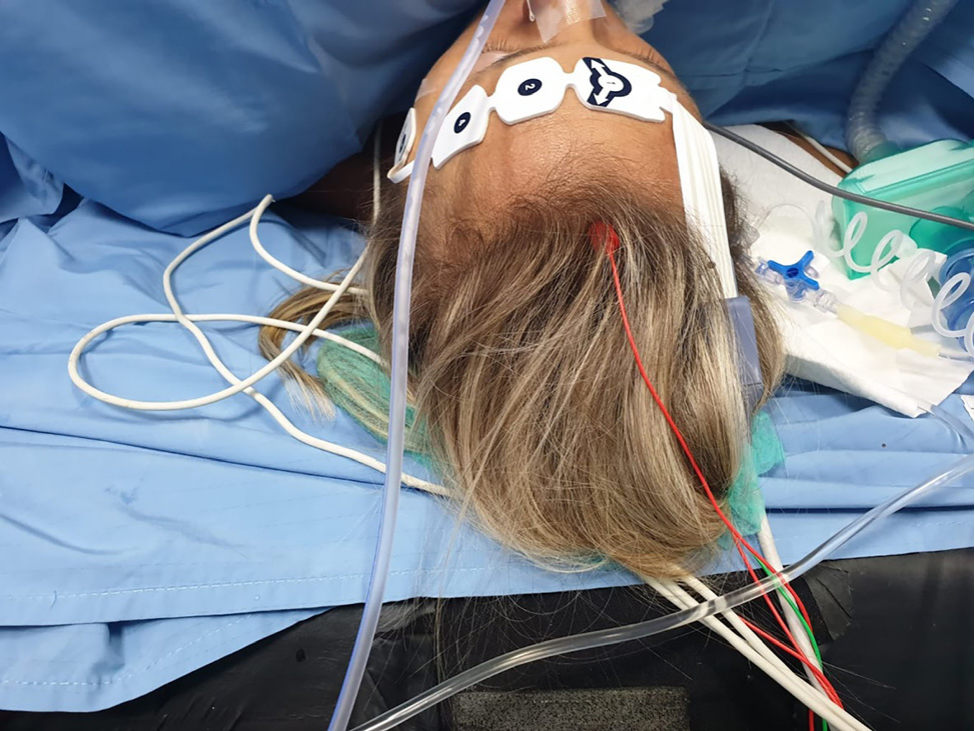
Transcranial electrical stimulation.

Medtronic E4 NIM Eclipse neuromonitoring system (Medtronic, Minneapolis, MN) is used for neuromonitoring. Neurostimulation with a bipolar probe (Figure 1) is performed during posterior rectal dissection, when separating the parietal and the visceral pelvic fascia at the level of the sacral promontory (hypogastric nerves), during anterolateral rectal dissection close to the lateral ligaments (inferior hypogastric plexus), and during anterior rectal dissection at the lateral edge of the rectovaginal fascia (urogenital neurovascular bundles). The currents applied range from 5 to 10 mA, and the frequency is 2 Hz, and stimulation lasted 5–20 s. The neuromonitoring paradigm consists of lower extremity and pudendal nerve somatosensory evoked potentials (SSEPs), transcranial motor evoked potential (TCeMEPs), and spontaneous electromyography (EMG) (free-run EMG) recorded from the external anal sphincter (EAS), bilateral bulbocavernosus (BULB), and lower extremity muscles bilaterally. Moreover, the lumbosacral plexus, and in particular the pudendal (PUD) nerve, is monitored by direct current stimulation of the nerves. Electroencephalography (EEG) is performed to evaluate the depth of anesthesia. SSEPs of lower extremity are obtained by electrical stimulation of the tibialis nerve on the right and left medial malleoli (Figure 2), with impulses of 250-μs duration, 3.79-Hz frequency, and cortical derivation from Cz’–Fpz according to the international 10–20 system (SI 10/20). PUD nerve SSEP is obtained through electrical stimulation of the PUD nerve over the clitoris with impulses of 200 μs duration, 3.79 Hz and cortical derivation from Cz’–Fpz (SI 10/20). TCeMEP is obtained through transcranial electrical stimulation (Figure 3), C2–C1, and reverse (SI 10/10) with train stimulus of seven impulses, 50-μs duration, 2-ms inter-stimulus interval (ISI). Signals were recorded through subcutaneous needles inserted in the external oblique muscle, iliopsoas, gracilis muscle, rectus femoris, tibialis anterior, hallux abductor, EAS, and BULB. Free-run EMG is continuously recorded from the nerve roots deriving from the aforementioned muscles for the TCeMEP. Cortical derivation Cz’–Fpz is used for EEG. Corkscrew or subdermal needle electrodes are used for scalp and muscle derivation.

Before starting the pelvic dissection, baseline registration of the SSEPs and TCeMEPs must be recorded.

## Results

IONM, permitting to identify, during CRS, eventual variations of the electrophysiological context, helps the surgeon to identify the correct plane of dissection. During closing, the analysis of the motor evoked potential and SSEPs and of the electrophysiological tests permits to confirm the preservation of pelvic nerves.

## Discussion

CRS plus HIPEC improved the prognosis of PSMs [1] despite the percentage of complications being underestimated [8]. The QOL is obviously also affected by such aggressive surgery, albeit in a limited way [9]. Pelvic peritonectomy can be responsible of anorectal and urogenital dysfunctions, correlated with nerve damage, virtually separating the parietal and the visceral pelvic fascia at the level of the sacral promontory (hypogastric nerves), during anterolateral rectal dissection close to the lateral ligaments (inferior hypogastric plexus) and during anterior rectal dissection at the lateral edge of rectovaginal fascia (urogenital neurovascular bundles). In literature, there are few reports about post-CRS anorectal and urogenital dysfunctions; Zenasni et al. [3] reported that 76.8% of women survivors had sexual difficulties and that men also reported some problems with sexual functioning (median = 33.3); the authors conclude that sexual dysfunctions could be related to pelvic surgery and to the peritoneal metastases knowledge of the patient himself. In the light of these considerations, to improve the QOL of patients submitted to CRS plus HIPEC, it is important to not only cure the psychological aspect but also reduce the consequences of the pelvic innervation related to the pelvic peritonectomy. For this reason, based on the well-coded experiences in rectal surgery [4], [10], [11], we decided to apply IONM to CRS plus HIPEC. We retain that IONM is technically feasible during peritonectomy and that it can help preserve pelvic nerves. We think that this technical modification could have a substantial value because it provides the surgeon with a direct feedback of whether the plane of dissection is close to the pelvic autonomic nerves. Obviously, its definitive value remains to be elucidated.
